# Increased expression of stathmin and elongation factor 1α in precancerous nodules with telomere dysfunction in hepatitis B viral cirrhotic patients

**DOI:** 10.1186/1479-5876-12-154

**Published:** 2014-05-31

**Authors:** Ei Yong Ahn, Jeong Eun Yoo, Hyungjin Rhee, Myung Soo Kim, Junjeong Choi, Jung Eun Ko, Jee San Lee, Young Nyun Park

**Affiliations:** 1 Department of Pathology, Yonsei University College of Medicine, 250 Seongsan-ro, Seodaemun-gu, Seoul, South Korea; 2 Brain Korea 21 PLUS Project for Medical Science, Seoul, South Korea; 3 Department of Pathology, Wonju Christian Hospital, Wonju, South Korea; 4 Department of Surgery, Yonsei University College of Medicine, Seoul, South Korea; 5 Severance Biomedical Science Institute, Yonsei University College of Medicine, Seoul, South Korea; 6 Integrated Genomic Research Center for Metabolic Regulation, Yonsei University College of Medicine, Seoul, South Korea

**Keywords:** Stathmin, Elongation factor 1α, Telomere dysfunction, Hepatocarcinogenesis, Dysplastic nodule, Hepatocellular carcinoma, Hepatitis B virus

## Abstract

**Background:**

Telomere dysfunction is important in carcinogenesis, and recently, stathmin and elongation factor 1α (EF1α) were reported to be up-regulated in telomere dysfunctional mice.

**Methods:**

In the present study, the expression levels of stathmin and EF1α in relation to telomere length, telomere dysfunction-induced foci (TIF), γ-H2AX, and p21^WAF1/CIP1^ expression were assessed in specimens of hepatitis B virus (HBV)-related multistep hepatocarcinogenesis, including 13 liver cirrhosis specimens, 14 low-grade dysplastic nodules (DN), 17 high-grade DNs, and 14 hepatocellular carcinomas (HCC). Five normal liver specimens were used as controls. TIF were analyzed by telomere fluorescent in situ hybridization (FISH) combined with immunostaining, while the protein expressions of stathmin, EF1α, γ-H2AX, and p21^WAF1/CIP1^ were detected by immunohistochemistry.

**Result:**

The expressions of stathmin and EF1α gradually increased as multistep hepatocarcinogenesis progressed, showing the highest levels in HCC. Stathmin mRNA levels were higher in high-grade DNs than normal liver and liver cirrhosis, whereas EF1α mRNA expression did not show such a difference. The protein expressions of stathmin and EF1α were found in DNs of precancerous lesions, whereas they were absent or present at very low levels in normal liver and liver cirrhosis. Stathmin histoscores were higher in high-grade DNs and low-grade DNs than in normal liver (all, *P* < 0.05). EF1α histoscores were higher in high-grade DNs than in normal liver and liver cirrhosis (all, *P* < 0.05). Stathmin mRNA levels and histoscores, as well as EF1α histoscores (but not mRNA levels), were positively correlated with telomere shortening and γ-H2AX labeling index (all, *P* < 0.05). EF1α histoscores were also positively correlated with TIF (*P* < 0.001). Significantly greater inactivation of p21^WAF1/CIP1^ was observed in low-grade DNs, high-grade DNs, and HCC, compared to liver cirrhosis (all, *P* < 0.05). p21^WAF1/CIP1^ labeling index was inversely correlated with TIF, stathmin mRNA level, and EF1α histoscore (all, *P* < 0.05).

**Conclusion:**

Stathmin and EF1α are suggested to be closely related to telomere dysfunction, DNA damage, and inactivation of p21^WAF1/CIP1^ in HBV-related multistep hepatocarcinogenesis. Accordingly, assessment of stathmin and EF1α levels as a reflection of telomere dysfunction may be helpful in evaluating the biological characteristics of precancerous hepatic nodules in hepatitis B viral cirrhotic patients.

## Background

Hepatocellular carcinoma (HCC) is reported as the fifth most common cancer worldwide and the third most common cause of cancer-related deaths [1]. Hepatocarcinogenesis is a multistep process, and has been characterized as the progressive accumulation of genetic and molecular changes in chronic liver disease, which leads to the production of monoclonal populations of dysplastic or transformed hepatocytes. Mutations that impair the DNA damage response pathways facilitate the survival of dysplastic cells with shortened and dysfunctional telomeres, leading to the proliferation of cells with dicentric chromosomes and the accumulation of genomic instability due to breakage-fusion-bridge cycles. In fact, chromosomal instability is characteristic of hepatitis B virus (HBV)-related hepatocarcinogenesis [2]. Liver cirrhosis, a soil for HCC, is characterized by an increase in dysplastic hepatocytes that form dysplastic foci or dysplastic nodules (DNs). Nevertheless, while DNs are considered precancerous lesions of HCC [3], various natural outcomes of DNs have been reported [4,5].

Previously, expression of stathmin and elongation factor 1α (EF1α) was reported to be up-regulated in telomere dysfunctional mice [6]. Stathmin, encoded by the human *STMN1* gene, is a major microtubule depolymerizing protein involved in cell cycle progression [7]. Stathmin directly interacts with soluble tubulin to form a complex that sequesters free tubulin and hinders the polymerization of microtubules [8]. In mitosis, stathmin is inactivated by phosphorylation to induce microtubule polymerization and the assembly of mitotic spindles; phosphorylated stathmin is reactivated by dephosphorylation, allowing cells to exit mitosis and enter a new interphase [9]. Stathmin, also known as oncoprotein 18, has been reported in several studies to be highly expressed in a wide variety of human cancers, including leukemia, breast, prostate, gastric, and liver cancer, suggesting that stathmin is a key molecule in tumorigenesis [10-13]. Moreover, stathmin overexpression in HCC reportedly increased the malignant potential thereof by regulating cell motility, cell migration, and cell proliferation [12]. Stathmin overexpression in HCC was also shown to be correlated with poor prognosis [14,15]. EF1α is a translational cofactor of eukaryotic protein synthesis that is important in the elongation of polypeptides. EF1α carries aminoacyl-tRNA to ribosomes and dissociates after correct codon-anticodon recognition by GTP hydrolysis [16]. Overexpression of EF1α in human and rodent cells was reported to be associated with increased cell proliferation, oncogenic transformation, and delayed cell senescence [17]. A previous functional study showed that EF1α interacts with p-Akt to control the activity of p-Akt and regulates the proliferation, survival, and motility of breast cancer cells [18]. Additionally, increased expression of EF1α was reported to be associated with the growth rate of an HCC cell line, but not with apoptosis [19].

The assessment of telomere dysfunction may be helpful in evaluating the biological characteristics of hepatic nodules in cirrhotic patients. However, systemic assessment of the expression levels of stathmin and EF1α in relation to telomere dysfunction, particularly telomere length and telomerase dysfunction induced foci (TIF), has not been conducted in defined lesions of human multistep hepatocarcinogenesis, including DNs. Accordingly, the present study attempted to evaluate and compare the expression levels of stathmin and EF1α in relation to telomere length, TIF, γ-H2AX, and p21^WAF1/CIP1^ expression in human HBV-associated multistep hepatocarcinogenesis.

## Materials and methods

### Tissue samples and pathological examination

A total of 58 liver specimens from 24 patients were investigated, including 13 cases of liver cirrhosis, 14 cases of low-grade DNs, 17 cases of high-grade DNs, and 14 cases of HCC. The specimens were obtained from 19 men and five women whose ages ranged from 32 to 63 years (51 ± 7.8 years, mean ± SD). They were all serum HBsAg-positive and anti-HCV-negative. The clinicopathological features of the patients are summarized in Table 1. Large nodules, measuring at least 0.5 cm at their largest dimension, that were apparently distinct from the surrounding cirrhotic parenchyma, in terms of their color, texture, and degree of bulging at the cut surface; HCCs; and corresponding non-neoplastic liver tissue were collected. Half of each nodule was fixed in 10% buffered formalin, routinely processed, and embedded in paraffin for histological examination. The remaining half of each nodule was snap-frozen in liquid nitrogen and stored at -80°C until ready for use. Hepatocellular nodules were evaluated according to the criteria proposed by the International Consensus Group for Hepatocellular Neoplasia [3]. Differentiation of HCC was determined on the basis of Edmonson and Steiner classification [20]. For comparison, non-neoplastic liver tissues were obtained from 5 patients with metastatic carcinoma. Control subjects were negative for hepatitis virus and showed relatively normal liver histology. The ages of the controls ranged from 42 to 74 years (61 ± 13.9 years). Fresh frozen specimens were obtained from the Liver Cancer Specimen Bank (part of the National Research Bank Program, Korea Science and Engineering Foundation, Ministry of Science and Technology). This study was approved by the Institutional Review Board of Severance Hospital, Yonsei University College of Medicine, and the requirement for informed consent was waived.

**Table 1:** Clinical and pathological findings of the patients

**Case no**	**Age**	**Sex**	**Etiology**	**Pathological diagnosis**^**a**^
1	44	F	HBV	LC, LGDN, HGDN, HGDN, HCC (2)
2	52	M	HBV	LC, LGDN, HGDN, HGDN, HGDN, HGDN, HCC (2), HCC (2)
3	60	F	HBV	LC, HGDN, HGDN, HCC (1)
4	48	M	HBV	LC, HGDN, HGDN, HCC (1)
5	49	M	HBV	LC, LGDN, LGDN, LGDN, LGDN, LGDN, LGDN, HCC (3)
6	53	F	HBV	LGDN, HGDN
7	53	M	HBV	LGDN, HGDN
8	45	M	HBV	LC, HGDN
9	60	M	HBV	LC, HGDN
10	59	M	HBV	LC, HGDN
11	32	F	HBV	LC, LGDN
12	48	M	HBV	LC, LGDN, LGDN
13	59	M	HBV	LC, HCC (1),
14	56	M	HBV	HCC (3)
15	41	M	HBV	HCC (3)
16	43	M	HBV	HCC (3)
17	40	M	HBV	HCC (2)
18	60	M	HBV	HCC (2)
19	54	F	HBV	HCC (2)
20	49	M	HBV	HCC (2)
21	51	M	HBV	LGDN
22	63	M	HBV	HGDN, HGDN
23	48	M	HBV	LC
24	48	M	HBV	LC

M, male; F, female; HBV, hepatitis B virus; LGDN, low-grade dysplastic nodule; HGDN, high-grade dysplastic nodule; HCC, hepatocellular carcinoma.

^a^HCC grading according to the Edmondson and Steiner classification is in parentheses.

### Total RNA extraction and real-time quantitative RT-PCR

Total RNA was isolated from human fresh frozen liver tissue samples using TRIzol reagent (Invitrogen, Carlsbad, CA, USA). The transcription thereof into cDNA was performed using a High Capacity RNA-to-cDNA kit (Applied Biosystems, Foster City, CA, USA). The following TaqMan Gene Expression Assays were purchased from Applied Biosystems: EF1α (Hs00265885_g1), stathmin (Hs01027516_g1), and GAPDH (Hs99999905_m1). All measurements were performed in triplicate. The relative expression levels of target mRNAs were normalized to GAPDH mRNA levels.

PCR was performed in triplicate for each cDNA sample using the ABI PRISM 7300 Sequence Detection System (Applied Biosystems). Dual-labeled FAM probes containing a 5′-fluorescent reporter and a 3′- quencher were used to conduct the PCR experiments.

### Immunohistochemical analysis of stathmin, EF1α, p21^WAF1/CIP1^, and γ-H2AX

Immunohistochemical staining was performed to detect the expression levels of stathmin, EF1α, p21^WAF1/CIP1^, and γ-H2AX, as previously described [2]. Details on the antibodies used and antigen-retrieval conditions are summarized in Additional file 1: Table S1.

The staining intensities of stathmin and EF1α were graded on a scale of 0–3 (0, negative; 1, weakly positive; 2, moderately positive; and 3, strongly positive), and the extent of distribution was rated on a scale of 0–4 (0, positive in <5% of cells; 1, 5-25%; 2, 26-50%; 3, 51-75%; and 4, 76 ~ 100%). Histoscore was defined as the sum of the intensity and distribution scores. For interpretation of the immunohistochemical stain results for γ-H2AX, dark brown stained nuclei were counted as being positive for antibody, and labeling indices were determined as follows: (number of positive hepatocytic nuclei in five randomly selected fields at × 400 magnification)/(total number of hepatocytic nuclei) × 100%.

### Telomere terminal restriction fragment length analysis

Telomere terminal restriction fragments were measured as previously described [2]. Briefly, telomere length was measured by Southern blotting. Two μg of digested DNA was separated on 0.7% agarose gel. Hybridization was carried out with 3′-end DIG-labeled d(TTAGGG)_4_ (Roche Molecular Biochemicals, Mannheim, Germany) and detected as recommended by the manufacturer. The resulting X-ray film was scanned with a luminescent image analyzer (Fujifilm, Tokyo, Japan), and the telomere signals in each lane were quantified as a grid object, defined as a single column with 25 rows, using Image Gauge Software 2.54 (Fujifilm).

### Telomere fluorescent in situ hybridization (FISH) combined immunostaining for γ-H2AX

TIF were detected in representative sections of formalin-fixed paraffin-embedded tissues by staining for γ-H2AX and by telomere *in situ* hybridization using a telomere-specific peptide nucleic acid probe (Cy3-(CCCTAA)_3_; Panagene, Daejeon, Korea). Tissue sections were deparaffinized and then rehydrated with graded alcohol. After incubation in 0.2 N HCl for 20 min, slides were boiled in 10 mM citrate buffer, pH 6.0, for 15 min to retrieve antigens. Sections were then treated with protease solution (Abbott Molecular Inc, Des Plaines, IL, USA) and fixed. Sections were dehydrated in 100% ethanol for 5 min. After air-drying, slides were applied 10 μL of a telomere peptide nucleic acid probe mixture (70% formamide deionized, 5 mM Tris–HCl, pH 7.4, 1 mM MgCl_2_, 0.45 mM Citric acid, 4.1 mM NaHPO_4_, 0.1 μg telomere-specific peptide nucleic acid probe, 5% blocking reagent [Roche Molecular Biochemicals]), denatured at 80°C for 3 min, and hybridized at 30°C for 2 hrs. Slides were then washed sequentially with 0.6X SSC/70% formamide (90 mM NaCl, 9 mM Na-citrate [pH 7.0]; 3 × 15 min), 2X SSC (2 × 15 min), PBS (1 × 5 min), and PBST (PBS + 0.1% Tween 20:15 min), and then blocked with 5% BSA for 5 min. Immunostaining using primary polyclonal anti-γ-H2AX (1:500; Novus Biologicals, Littleton, CO, USA) and secondary Alexa Fluor 488-conjugated goat anti rabbit antibody (1:1,000; Invitrogen) was performed.

### Statistical analysis

Statistical analysis was conducted using SPSS (version 18.0.0; SPSS Inc., Chicago, IL, USA) and R package software (version 3.0.2; http://www.R-project.org), applying the Mann–Whitney test, Spearman’s correlation coefficient, linear model, and log linear model as deemed appropriate. Significance was set at *P* < 0.05 for all tests.

## Results

### Stathmin expression in HBV-related multistep hepatocarcinogenesis

**Figure 1:**
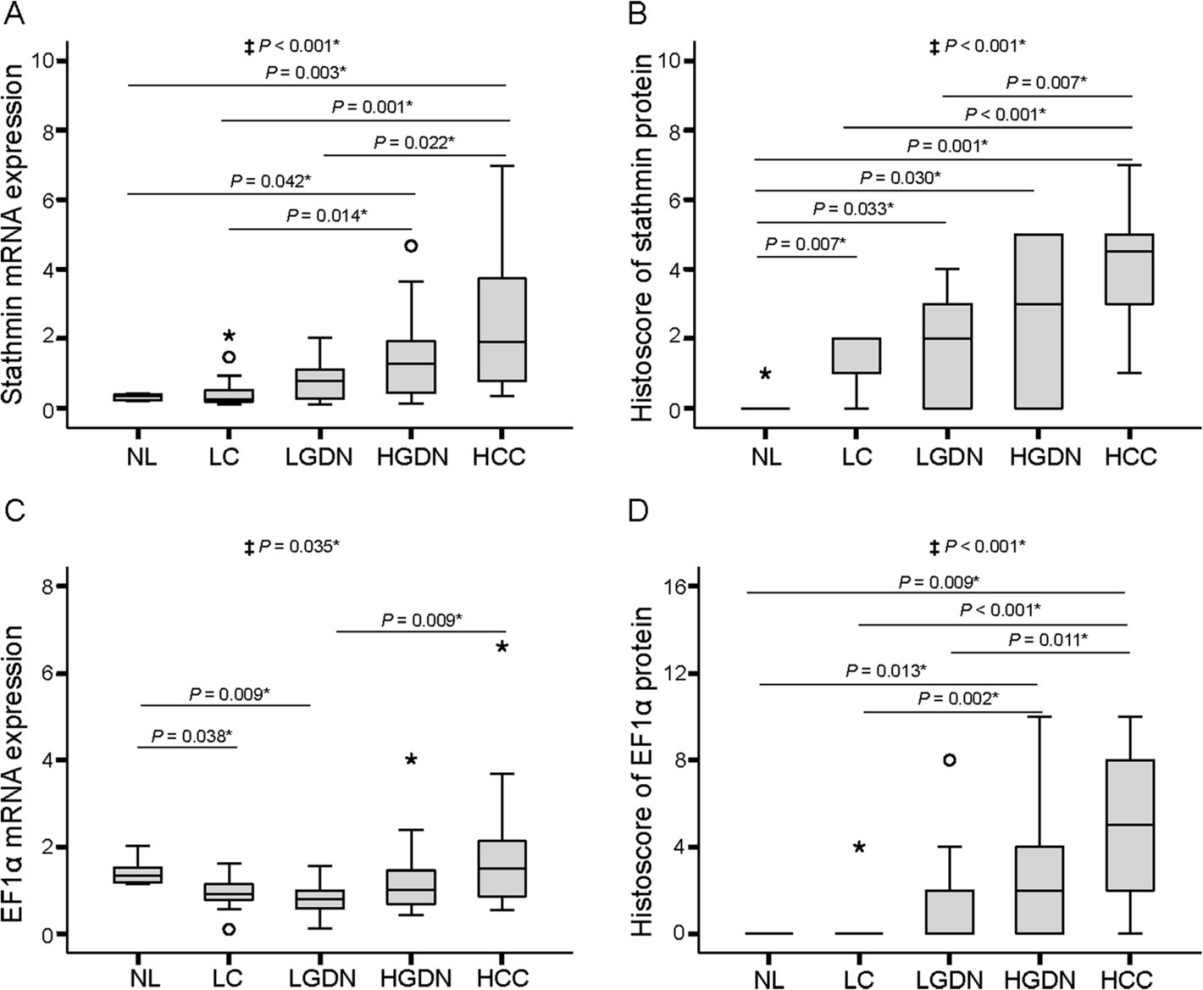
**Expression levels of stathmin and elongation factor 1α (EF1α) in HBV-related multistep hepatocarcinogenesis.** Box plots show expression levels of stathmin mRNA **(A)**, stathmin protein **(B)**, EF1α mRNA **(C)**, and EF1α protein **(D)**. ‡ Statistical significance (linear trend model, *P* < 0.05) *Statistical significance (*P* < 0.05). NL, normal liver; LC, liver cirrhosis; LGDN, low-grade dysplastic nodules; HGDN, high-grade dysplastic nodules; HCC, hepatocellular carcinoma.

The mRNA levels of stathmin were 0.3 ± 0.12 (mean ± SD) (range, 0.2 ~ 0.5) in normal liver, 0.5 ± 0.61 (0.1 ~ 2.1) in liver cirrhosis, 0.8 ± 0.57 (0.1 ~ 2.0) in low-grade DNs, 1.5 ± 1.29 (0.1 ~ 4.7) in high-grade DNs, and 2.3 ± 1.95 (0.4 ~ 7.0) in HCC. Stathmin mRNA levels gradually increased as multistep hepatocarcinogenesis progressed from normal liver, low-grade DNs, and high-grade DNs to HCC, which showed the highest level of expression and statistical significance (*P* for trend < 0.001) (Figure 1A). The differences in mRNA levels were statistically significant between normal liver and high-grade DNs, as well as between liver cirrhosis and high-grade DNs (all, *P* < 0.05). Stathmin mRNA levels in HCC were significantly higher than those in normal liver, liver cirrhosis, and low-grade DNs (all, *P* < 0.05).

**Figure 2:**
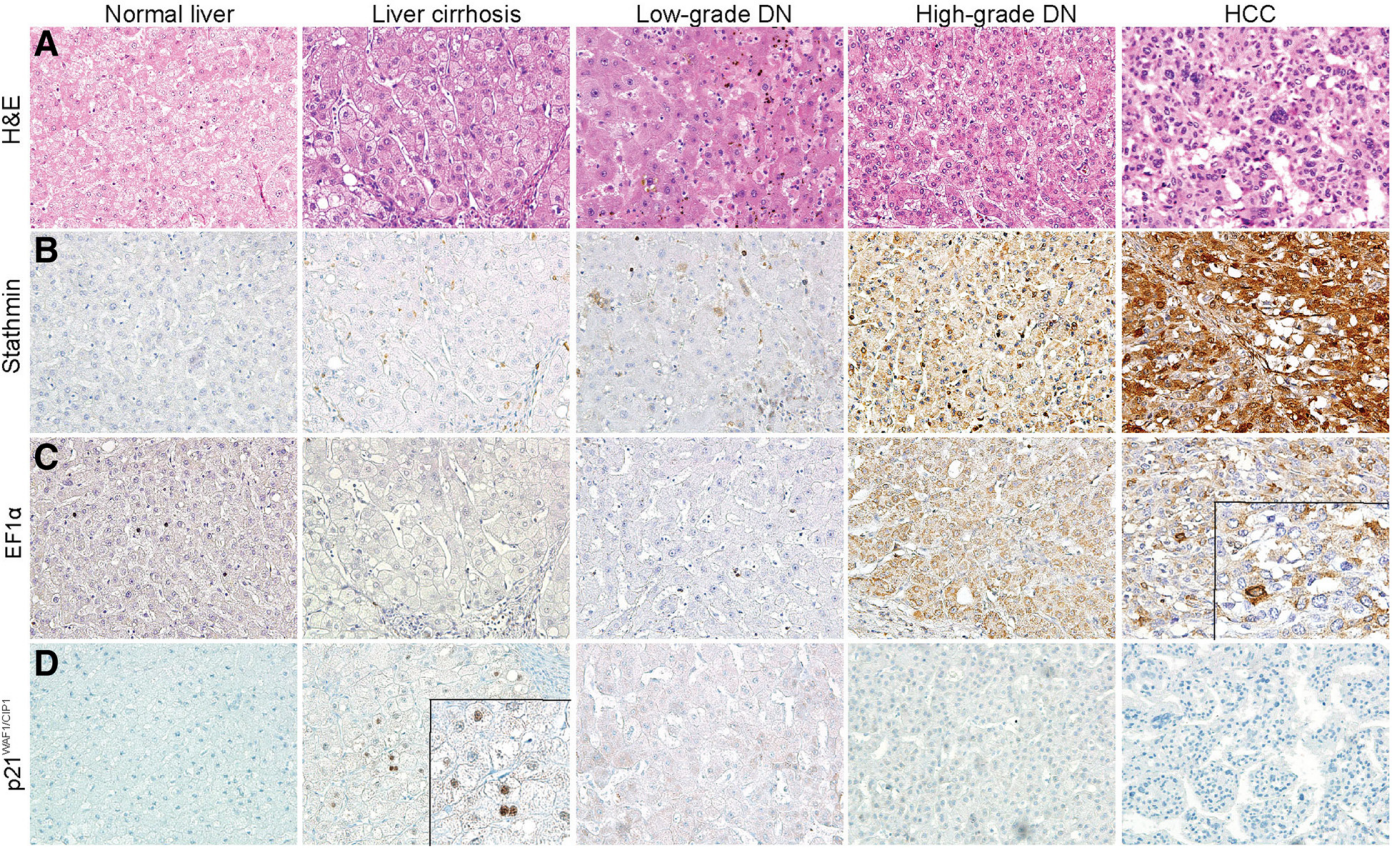
**Representative immunohistochemical features of stathmin, elongation factor 1α (EF1α) and p21**^**WAF1/CIP1 **^**in HBV-related multistep hepatocarcinogenesis.** Representative pathological features of normal liver (NL), liver cirrhosis (LC), low-grade dysplastic nodules (LGDN), high-grade dysplastic nodules (HGDN), and hepatocellular carcinoma (HCC) are presented in the upper panel **(A)**. Immunohistochemical findings of stathmin **(B)**, EF1α **(C)** and p21^WAF1/CIP1 ^**(D)** for each type of lesion are shown, respectively (Original magnification × 200). Inset, higher power magnification of EF1α and p21^WAF1/CIP1^ (×400).

Similarly, the expression of stathmin protein also gradually increased as multistep hepatocarcinogenesis progressed towards HCC (*P* for trend < 0.001) (Figure 1B, Figure 2B). Most normal liver specimens showed no expression of stathmin protein, except for one case that showed weak expression of histoscore grade 1. The mean histoscores for stathmin were 1.3 ± 0.63 in liver cirrhosis, 2.0 ± 1.57 in low-grade DNs, 2.6 ± 2.12 in high-grade DNs, and 4.1 ± 1.77 in HCC. Stathmin histoscores were significantly different between liver cirrhosis and HCC, as well as between low-grade DNs and HCC; moreover, stathmin histoscores were significantly greater in liver cirrhosis, low-grade DNs, high-grade DNs, and HCC than in normal liver (all, *P* < 0.05).

### EF1α expression in HBV-related multistep hepatocarcinogenesis

The mRNA levels of EF1α were 2.9 ± 0.71 (mean ± SD) (range, 2.3 ~ 4.0) in normal liver, 1.9 ± 0.83 (0.2 ~ 3.2) in liver cirrhosis, 1.6 ± 0.74 (0.3 ~ 3.1) in low-grade DNs, 2.5 ± 1.76 (0.9 ~ 8) in high-grade DNs, and 3.9 ± 3.19 (1.1 ~ 13.2) in HCC (Figure 1C). HCC showed the highest level of EF1α mRNA expression, which was significantly higher than that in low-grade DNs (*P* = 0.009), whereas liver cirrhosis and low-grade DNs showed lower levels of EF1α mRNA expression than normal liver (*P* < 0.05 for both). The mRNA expression of EF1α showed significant increases as multistep hepatocarcinogenesis progressed towards HCC (*P* for trend = 0.035).

EF1α protein was not expressed in normal liver, but was detected in dysplastic and HCC cells (Figure 2C). The histoscores of EF1α protein were 0.2 ± 0.55 (mean ± SD) (range, 0 ~ 2) in liver cirrhosis, 0.7 ± 1.14 (0 ~ 4) in low-grade DNs, 1.3 ± 1.36 (0 ~ 5) in high-grade DNs, and 2.4 ± 1.87 (1 ~ 7) in HCC (Figure 1D), and gradually increased from liver cirrhosis, low-grade DNs, high-grade DNs to HCC with statistical significance (*P* for trend < 0.001). HCC showed the highest EF1α histoscore, which was significantly higher than that for low-grade DNs, liver cirrhosis and normal liver (all, *P* < 0.05). EF1α histoscores were also significantly greater in high-grade DNs than in liver cirrhosis (*P* = 0.002) and normal liver (*P* = 0.013). The histoscores of EF1α and stathmin were shown to be positively correlated (*P* < 0.001, R = 0.443), whereas EF1α and stathmin mRNA levels showed no significant correlation (*P* = 0.558, R = 0.075).

### Telomere length and its relationship with stathmin and EF1α expression in HBV-related multistep hepatocarcinogenesis

**Figure 3:**
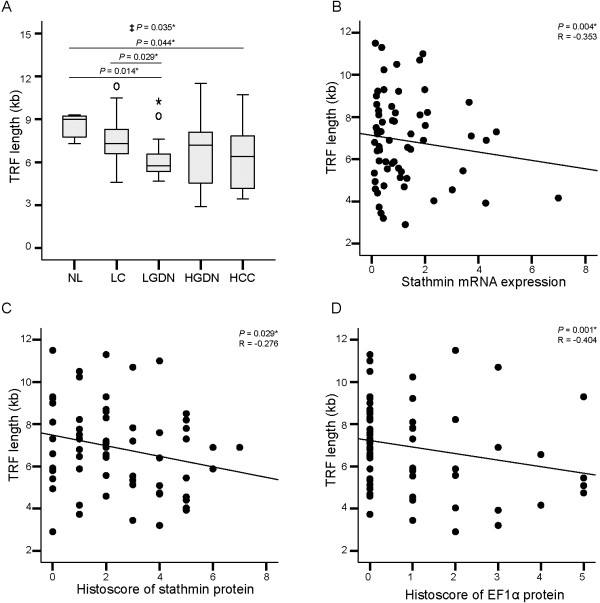
**Telomere length and the correlations thereof with stathmin and elongation factor 1α (EF1α) expression in HBV-related multistep hepatocarcinogenesis. A**. Telomere terminal restriction fragment (TRF) length in normal liver (NL), liver cirrhosis (LC), low-grade dysplastic nodules (LGDN), high-grade dysplastic nodules (HGDN), and hepatocellular carcinoma (HCC). **B-C**. Correlation between TRF length and stathmin mRNA **(B)** and stathmin protein levels **(C)**. **D**. Correlation between TRF length and EF1α protein levels. ‡ Statistical significance (linear trend model, *P* < 0.05). *Statistical significance (*P* < 0.05).

Telomere length gradually decreased as multistep hepatocarcinogenesis progressed towards HCC, with statistical significance (*P* for trend = 0.032); part of this data was previously reported [2]. Telomere lengths were 8.5 ± 0.92 (mean ± SD) (range, 7.3 ~ 9.3) in normal liver, 7.6 ± 1.81 (4.6 ~ 11.3) in liver cirrhosis, 6.4 ± 1.63 (4.7 ~ 10.2) in low-grade DNs, 6.8 ± 2.54 (2.9 ~ 11.5) in high-grade DNs, and 6.3 ± 2.19 (3.4 ~ 10.7) in HCC (Figure 3A).

Next, the associations of telomere length with stathmin and EF1α expression were evaluated. Both mRNA levels and histoscores for stathmin showed significant negative correlations with telomere length (Figure 3B and C) (*P* = 0.004, R = -0.353 and *P* = 0.029, R = -0.276, respectively). EF1α histoscore showed a significant negative correlation with telomere length (*P* = 0.001, R = -0.404) (Figure 3D), whereas EF1α mRNA level showed no significant correlation with telomere length (*P* = 0.719, R = -0.046) (Additional file 2: Figure S1A).

### γ-H2AX expression and its relationship with stathmin and EF1α expression and telomere length in HBV-related multistep hepatocarcinogenesis

**Figure 4:**
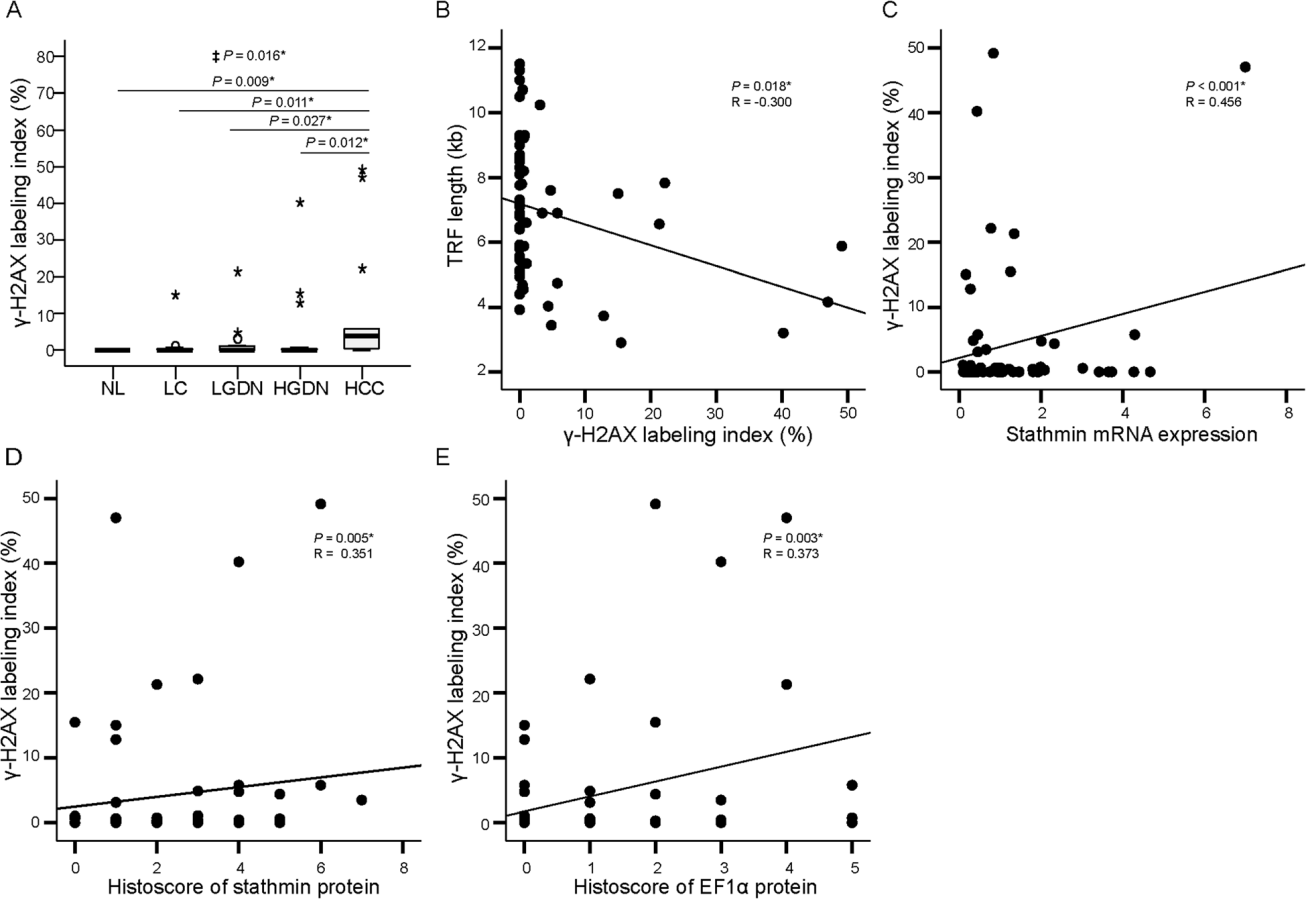
**Expression of γ-H2AX and its correlation with telomere length, stathmin and elongation factor 1α (EF1α) expression in HBV-related multistep hepatocarcinogenesis. A**. γ-H2AX labeling index in normal liver (NL), liver cirrhosis (LC), low-grade dysplastic nodules (LGDN), high-grade dysplastic nodules (HGDN), and hepatocellular carcinoma (HCC). ‡ Statistical significance (linear trend model, *P* < 0.05). **B**. Correlation between γ-H2AX labeling index and telomere length. **C-D**. Correlation between γ-H2AX labeling index and stathmin mRNA **(C)** and stathmin protein level **(D)**. **E**. Correlation between γ-H2AX labeling index and EF1α protein levels **(F)**. *Statistical significance (*P* < 0.05).

To evaluate DNA damage in multistep hepatocarcinogenesis, immunohistochemical analysis of γ-H2AX was performed. The expression of γ-H2AX protein was detected in the nuclei of dysplastic and neoplastic cells. The γ-H2AX labeling indices were 1.3 ± 4.13 (mean ± SD) (range, 0 ~ 15.0) in liver cirrhosis, 2.4 ± 5.67 (0 ~ 21.3) in low-grade DNs, 4.1 ± 10.42 (0 ~ 40.2) in high-grade DNs, and 10.3 ± 17.00 (0 ~ 49.0) in HCC (Figure 4A). γ-H2AX labeling index showed a gradual increase from liver cirrhosis, low-grade DNs, and high-grade DNs to HCC with statistical significance (*P* for trend = 0.016) . γ-H2AX labeling index in HCC was significantly higher than that in normal liver, liver cirrhosis, low-grade DNs, and high-grade DNs (all, *P* < 0.05). Additionally, γ-H2AX labeling index showed a significant negative correlation with telomere length (*P* = 0.018, R = -0.300); higher expression of γ-H2AX labeling index was found in lesions with shorter telomere lengths (Figure 4B).

The associations between γ-H2AX labeling index and the expression levels of stathmin and EF1α were further analyzed. γ-H2AX labeling index showed a significant positive correlation with both mRNA levels and histoscores of stathmin (*P* < 0.001, R = 0.456 and *P* = 0.005, R = 0.351, respectively), indicating that an increase in stathmin expression is associated with DNA damage (Figure 4C and D). For EF1α expression, histoscores of EF1α protein showed a positive correlation with γ-H2AX index (*P* < 0.003, R = 0.373) (Figure 4D); however, we observed no significant correlations between EF1α mRNA level and γ-H2AX labeling index (*P* = 0.555, R = -0.076) (Additional file 2: Figure S1B).

### Telomere dysfunction induced foci (TIF) and their relationship with stathmin and EF1α expression in HBV-related multistep hepatocarcinogenesis

**Figure 5:**
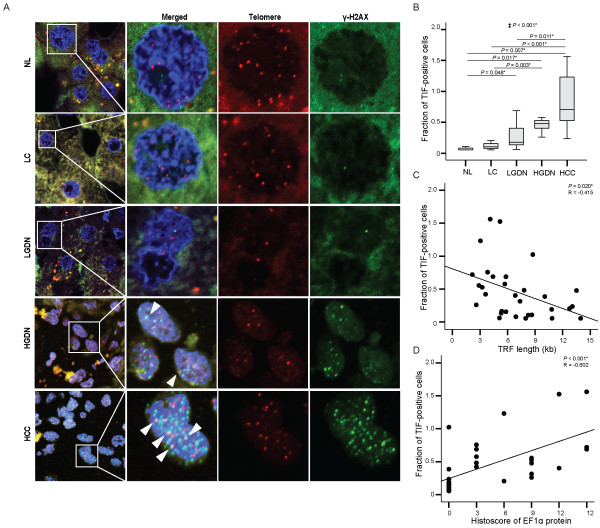
**Telomere dysfunctional induced foci (TIF) in HBV-related multistep hepatocarcinogenesis and the correlations thereof with stathmin and elongation factor 1α (EF1α) expression. A**. Representative features of colocalization of γ-H2AX and telomeric DNA in defined lesions of human multistep hepatocarcinogenesis. TIF are indicated by colored arrowheads: blue, DAPI; green, γ-H2AX; red, telomeres; yellow, TIF. **B**. Box plots show fractions of TIF-positive cells in normal liver (NL), liver cirrhosis (LC), low-grade dysplastic nodules (LGDN), high-grade dysplastic nodules (HGDN), and hepatocellular carcinoma (HCC). ‡ Statistical significance (linear trend model, *P* < 0.05). **C**. Correlation between TIF and TRF length. **D**. Correlation between TIF and histoscore EF1α. *Statistical significance (*P* < 0.05).

In order to determine the fractions of TIF during human multistep hepatocarcinogenesis, we performed telomere FISH combined with immunostaining for anti-γ-H2AX to detect colocalization of telomeres and γ-H2AX foci (Figure 5A). A gradual increase of TIF was observed as multistep hepatocarcinogenesis progressed towards HCC with statistical significance (*P* for trend < 0.01) (Figure 5B). Normal liver showed the lowest average number of TIF, 0.1 ± 0.30 (mean ± SD) (range, 0.06 ~ 0.16), and the number of TIF gradually increased as multistep hepatocarcinogenesis progressed from normal liver, liver cirrhosis, low-grade DNs, and high-grade DNs to HCC (liver cirrhosis; 0.1 ± 0.06 [0.06 ~ 0.21], low-grade DNs; 0.3 ± 0.23 [0.06 ~ 0.69], high-grade DNs; 0.4 ± 0.12 [0.26 ~ 0.76], HCC; 0.8 ± 0.48 [0.24 ~ 1.56]). HCC showed a significantly higher number of TIF, compared to normal liver, liver cirrhosis, and low-grade DNs (all, *P* < 0.05); high-grade DNs showed a significantly higher number of TIF, compared to normal liver and liver cirrhosis (all, *P* < 0.05); and low-grade DNs showed a significantly higher number of TIF than normal liver (*P* = 0.048). We further analyzed the relationships between TIF and telomere length, as well as TIF and the protein expression levels of stathmin and EF1α. TIF showed a significant negative correlation with telomere length (*P* = 0.020, R = -0.415, Figure 5C). EF1α histoscore showed a significant positive correlation with TIF, suggesting that higher EF1α expression is associated with a greater number of TIF, indicating increased telomere dysfunction (*P* < 0.001, R = 0.602) (Figure 5D). Stathmin histoscore, however, showed no significant correlation with TIF (*P* = 0.326, R = 0.182) (Additional file 2: Figure S1C). We also observed no significant correlations between TIF and the mRNA levels of both stathmin and EF1α (*P* = 0.360, R = 0.170 and *P* = 0.263, R = 0.205, respectively).

### p21^WAF1/CIP1^ labeling index and its relationship with TIF, and stathmin and EF1α expression in HBV-related multistep hepatocarcinogenesis

**Figure 6:**
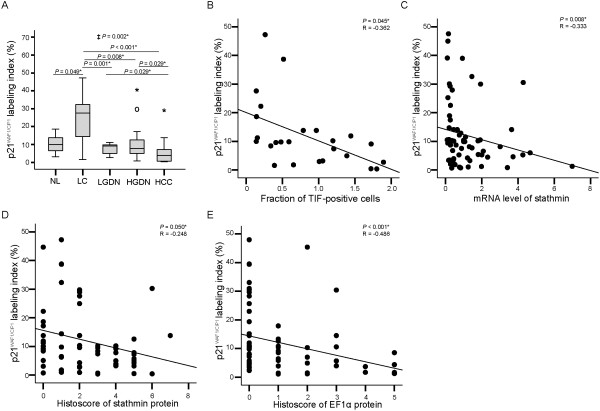
**p21**^**WAF1/CIP1 **^**labeling index in HBV-related multistep hepatocarcinogenesis and the correlations thereof with stathmin and elongation factor 1α (EF1α) expression. A**. Box plots show p21^WAF1/CIP1^ labeling index in normal liver (NL), liver cirrhosis (LC), low-grade dysplastic nodules (LGDN), high-grade dysplastic nodules (HGDN), and hepatocellular carcinoma (HCC). ‡ Statistical significance (linear trend model, *P* < 0.05). **B**. Correlations between p21^WAF1/CIP1^ labeling index and TIF. **C-D**. Correlations between p21^WAF1/CIP1^ labeling index and stathmin mRNA **(C)** and stathmin protein levels **(D)**. **E**. Correlations between p21^WAF1/CIP1^ labeling index and EF1α protein levels. *Statistical significance (*P* < 0.05).

The expression of p21^WAF1/CIP1^ protein was investigated in multistep hepatocarcinogenesis; part of the data was previously reported [2]. The p21^WAF1/CIP1^ labeling indices showed gradual statistically significant decreases as multistep hepatocarcinogenesis progressed towards HCC (*P* for trend = 0.002) (Figure 2D and Figure 6A).

The p21^WAF1/CIP1^ labeling indices were 10.4 ± 6.09 (mean ± SD) (range, 3.1 ~ 18.6) in normal liver, 24.9 ± 13.55 (1.6 ~ 47.2) in liver cirrhosis, 7.9 ± 2.90 (2.7 ~ 11.2) in low-grade DNs, 11.0 ± 11.11 (0.8 ~ 44.7) in high-grade DNs, and 5.9 ± 8.08 (0.4 ~ 30.2) in HCC. p21^WAF1/CIP1^ labeling index was highest in liver cirrhosis, which was significantly higher than that in normal liver, low-grade DNs, high-grade DNs, and HCC (all, *P <* 0.05). The p21^WAF1/CIP1^ labeling index of HCC was the lowest, and was significantly lower than that in low-grade DNs, high-grade DNs, and liver cirrhosis (all, *P <* 0.05).

p21^WAF1/CIP1^ labeling index showed a significant negative correlation with TIF (*P* = 0.045, R = -0.362) (i.e., higher TIF was correlated with lower p21^WAF1/CIP1^labeling index) (Figure 6B). Additionally, associations between p21^WAF1/CIP1^ labeling index and the expression levels of stathmin and EF1α were analyzed. Both stathmin mRNA levels and histoscores showed a negative correlation with p21^WAF1/CIP1^labeling index (Figure 6C and D) (*P* = 0.008, R = -0.333, and *P* = 0.05, R = -0.248, respectively) (i.e., higher stathmin expression was correlated with lower p21^WAF1/CIP1^labeling index). EF1α histoscore showed a significant negative correlation with p21^WAF1/CIP1^labeling index, as higher EF1α histoscores were correlated with lower p21^WAF1/CIP1^labeling index (*P* < 0.001, R = -0.486), whereas EF1α mRNA levels showed no significant correlation with p21^WAF1/CIP1^labeling index (*P* = 0.383, R = -0.112) (Figure 6E and Additional file 2: Figure S1D).

## Discussion

Telomere erosion occurs in proliferating cells with insufficient telomerase activity; thereby, critically short telomeres become dysfunctional and trigger apoptosis and/or senescence as a tumor suppressive mechanism. Meanwhile, mutations that impair the DNA damage response pathways allow for survival of cells with critically short telomeres. As a result, cells with dicentric chromosomes can proliferate and lead to the accumulation of genomic instability due to breakage-fusion-bridge cycles. In the present study, a gradual increase in shortened and dysfunctional telomeres was found as HBV-related multistep hepatocarcinogenesis progressed to HCC. Low-grade DNs showed a significantly higher number of TIF than normal liver; high-grade DNs exhibited a significantly greater number of TIF than liver cirrhosis and normal liver; and HCC showed the highest number of dysfunctional telomeres. Additionally, telomere length was inversely correlated with γ-H2AX labeling index, a DNA damage marker. These findings support that telomere dysfunction and DNA damage are important in the progression of HBV-related multistep hepatocarcinogenesis.

Stathmin was recently shown to be induced in response to telomere dysfunction and DNA damage in human aging and diseases [6]. Excessive stathmin activity was reported to generate chromosomal instability through blockage, subsequent to metaphase-to-anaphase transition, by decreasing the fidelity of chromosome segregation to spindle poles during anaphase in an *in vitro* study [21]. In the present study, stathmin expression at both the mRNA and protein level increased gradually as multistep hepatocarcinogenesis progressed from liver cirrhosis, low-grade DNs, and high-grade DNs to HCC, which showed the highest levels of expression. Interestingly, stathmin mRNA levels were significantly higher in high-grade DNs than in liver cirrhosis and normal liver and the histoscores of stathmin protein were higher in high-grade DNs and low-grade DNs than normal liver; stathmin expression was not detected in normal liver. Additionally, the protein and mRNA expression levels of stathmin were well correlated with γ-H2AX labeling index and telomere shortening; moreover, fractions of TIF-positive cells were also well correlated with telomere length shortening.

The primary function of EF1α is to transport aminoacyl-tRNA to ribosomes during protein translation. The overexpression of EF1α was previously reported to be associated with cell proliferation, oncogenic transformation, and delayed cell senescence in human and rodent cells [16,17]. As well, previous functional studies demonstrated that EF1α regulates the proliferation, survival, and motility of breast cancer cells [18] and induces higher proliferation capacity in undifferentiated HCC cell lines [19]. In the present study, EF1α protein expression gradually increased in human multistep hepatocarcinogenesis, and was highest in HCC. Interestingly, EF1α histoscore in HGDN was significantly higher than that in normal liver and liver cirrhosis. The expression levels of EF1α protein were well correlated with γ-H2AX labeling index and TIF, whereas they exhibited a significant inverse correlation with telomere length. Meanwhile, EF1α mRNA level showed no significant correlation with telomere length, γ-H2AX labeling index, TIF, or p21^WAF1/CIP1^ labeling index, and further investigation of a translational regulation mechanism for EF1α is needed. Notwithstanding, our data suggested that the expression of stathmin and EF1α arises in DNs as precancerous lesions and gradually increases along with progression of human hepatocarcinogenesis, suggesting that expression of stathmin and EF1α is induced by shortened and dysfunctional telomeres in B viral multistep hepatocarcinogenesis.

p21^WAF1/CIP1^ is a potent cyclin-dependent kinase inhibitor and the expression of this gene is controlled by the tumor suppressor protein p53, through which this protein mediates the p53-dependent cell cycle G1 phase arrest in response to a variety of stress stimuli [22]. In this study, p21^WAF1/CIP1^ was inactivated in low-grade DNs, high-grade DNs, and HCC, in contrast to its high expression in liver cirrhosis. Interestingly, p21^WAF1/CIP1^ labeling index showed a significant negative correlation with TIF, stathmin mRNA level, and the histoscores of EF1α protein in this study. Accordingly, the p21^WAF1/CIP1^ cell cycle check point was discerned to be a defense mechanism against damaged and transformed cells, triggered by dysfunctional telomeres in multistep hepatocarcinogenesis.

The natural history of DNs has not been fully clarified, and prospective studies conducted in large series of histologically proven non-neoplastic nodules detected by ultrasonography during surveillance programs of cirrhosis have demonstrated a wide evolutionary fate for such lesions. The hazard ratios of high-grade DNs, low-grade DNs, and large regenerative nodules for transformation to HCC were reported to be 16.8, 2.96, and 1.0, respectively [4,5]. However, it is difficult to predict the biological behavior of individual hepatic nodules, as some high-grade DNs remain stable for a long time period and a few of them even disappear. Therefore, the assessment of shortened and dysfunctional telomeres may be helpful to evaluating biological characteristics of hepatic nodules, although direct measurement of telomere dysfunction using telomere FISH combined immunostaining is very time consuming and labor intensive. Nevertheless, this study revealed that the expressions of stathmin and EF1α are good indicators of shortened and dysfunctional telomeres, and the evaluation of the expression levels thereof in conjunction with p21^WAF1/CIP1^ may prove helpful in characterizing the biological nature of hepatic nodules in B viral cirrhotic patients.

Here, we demonstrated for the first time that the expression of stathmin and EF1α are closely related to telomere dysfunction, DNA damage, and inactivation of p21^WAF1/CIP1^ in the defined lesions of HBV-related multistep hepatocarcinogenesis, including liver cirrhosis, low-grade DNs, high-grade DNs, and HCC. Accordingly, assessment of stathmin and EF1α levels as a reflection of telomere dysfunction may be helpful in evaluating the biological characteristics of precancerous hepatic nodules in hepatitis B viral cirrhotic patients.

## Competing interests

All authors have no competing interest to disclose.

## Author’s contributions

EY Ahn and JE Yoo designed the study and carried out experiments and analysis of data and drafted manuscript. J Choi analyzed some part of the data and participated in collection of human specimens. H Rhee, MS Kim, JE Ko and JS Lee participated in collection of human specimens. YN Park conceived of the study, participated in its design and coordination, and helped to draft the manuscript. All authors read and approved the final manuscript.

## Supplementary Material

Additional file 1: Table S1 Antibodies used in this study. Click here for file

Additional file 2: Figure S1 Correlation of stathmin and elongation factor 1α (EF1α) expression with telomere dysfunction and DNA damage in HBV-related multistep hepatocarcinogenesis. A-B. Scatter plots reveal a correlation between EF1α mRNA expression and telomere terminal restriction fragment (TRF) length (A) and γ-H2AX labeling index (B). C. Correlation between telomere dysfunction induced foci (TIF) and stathmin protein level. D. Scatter plot of a correlation between p21^WAF1/CIP1^ labeling index and EF1α mRNA expression. Click here for file
