# Supplementary material for: Increased expression of stathmin and elongation factor 1α in precancerous nodules with telomere dysfunction in hepatitis B viral cirrhotic patients
Source: J Transl Med. 2014 May 31;12:154. doi: 10.1186/1479-5876-12-154 (PMC4050101; doi:10.1186/1479-5876-12-154)
Supplement: Additional file 1: Table S1 — Antibodies used in this study. [file 1479-5876-12-154-S1.docx]

**Additional file 1: Table S1. Antibodies used in this study**

| Antibody | Source | Dilution | Antigen retrieval |
| --- | --- | --- | --- |
| Stathmin | Mouse monoclonal; Abcam, Cambridge, UK | 1:250 | Citrate buffer, pH 6.0 |
| EF1α | Mouse monoclonal; Proteintech Group, Chicago, IL | 1:100 | Citrate buffer, pH 6.0 |
| p21^WAF1/CIP1^ | Rabbit polyclonal; Dako, Glostrup, Denmark | 1:50 | Citrate buffer, pH 6.0 |
| γ-H2AX | Mouse monoclonal; Novus Biological, Littleton, CO | 1:100 | Citrate buffer, pH 6.0 |
