# Supplementary figures and images for: Increased expression of stathmin and elongation factor 1α in precancerous nodules with telomere dysfunction in hepatitis B viral cirrhotic patients
Source: J Transl Med. 2014 May 31;12:154. doi: 10.1186/1479-5876-12-154 (PMC4050101; doi:10.1186/1479-5876-12-154)

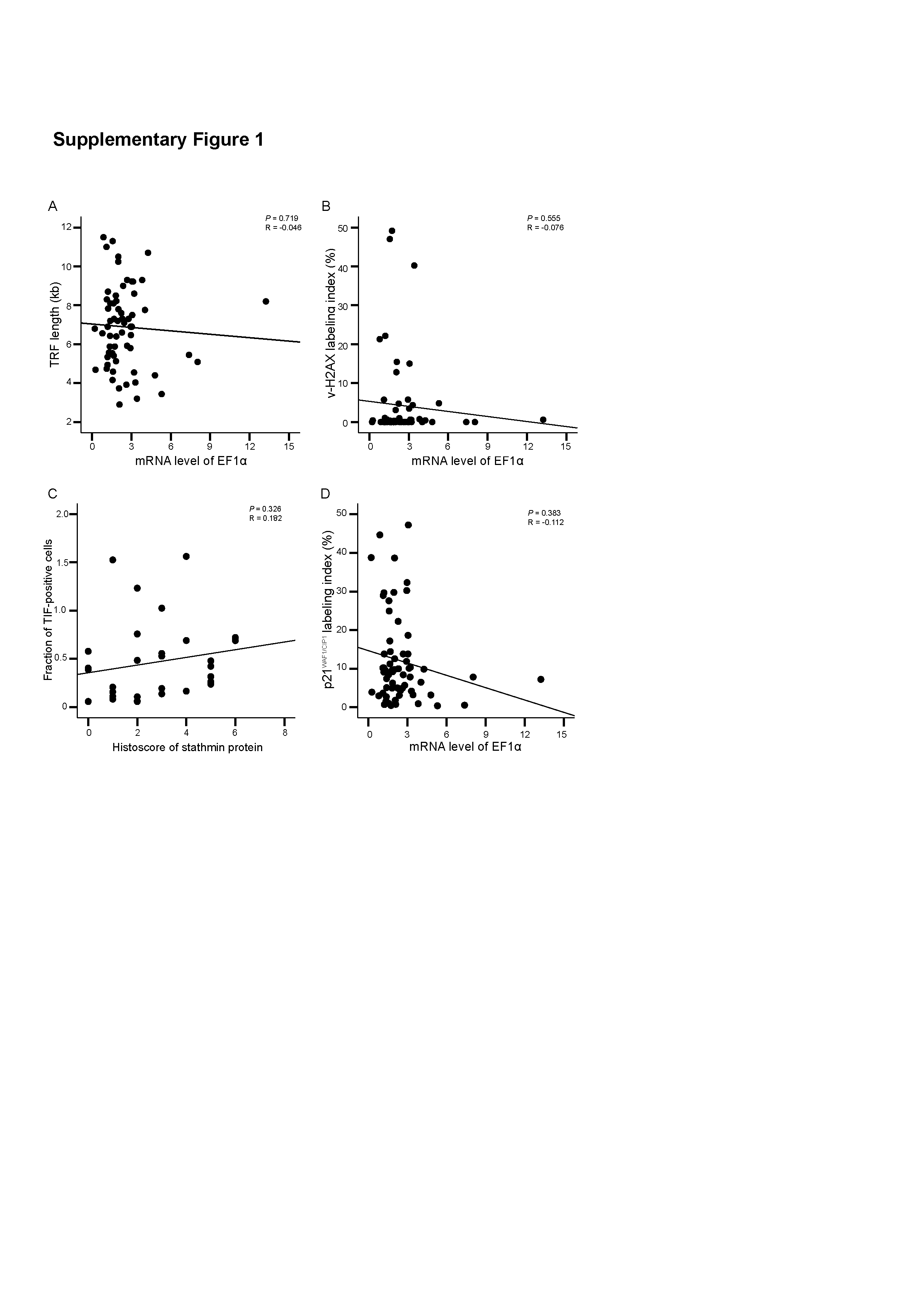

Supplement: Additional file 2: Figure S1 — Correlation of stathmin and elongation factor 1α (EF1α) expression with telomere dysfunction and DNA damage in HBV-related multistep hepatocarcinogenesis. A-B. Scatter plots reveal a correlation between EF1α mRNA expression and telomere terminal restriction fragment (TRF) length (A) and γ-H2AX labeling index (B). C. Correlation between telomere dysfunction induced foci (TIF) and stathmin protein level. D. Scatter plot of a correlation between p21WAF1/CIP1 labeling index and EF1α mRNA expression. [file 1479-5876-12-154-S2.tiff]
